# Replication of a Gene-Diet Interaction at *CD36*, *NOS3* and *PPARG* in Response to Omega-3 Fatty Acid Supplements on Blood Lipids: A Double-Blind Randomized Controlled Trial^☆^

**DOI:** 10.1016/j.ebiom.2018.04.012

**Published:** 2018-04-17

**Authors:** Ju-Sheng Zheng, Jiewen Chen, Ling Wang, Hong Yang, Ling Fang, Ying Yu, Liping Yuan, Jueping Feng, Kelei Li, Jun Tang, Mei Lin, Chao-Qiang Lai, Duo Li

**Affiliations:** a Institute of Basic Medical Sciences, Westlake Institute for Advanced Study, Westlake University, Hangzhou 310024, China; b Institute of Nutrition and Health, Qingdao University, Qingdao 266071, China; c Department of Food Science and Nutrition, Zhejiang University, Hangzhou 310058, China; d MRC Epidemiology Unit, University of Cambridge, Cambridge CB20QQ, UK; e Shanghai Ninth People's Hospital, Shanghai Jiao Tong University School of Medicine, Center for Specialty Strategy Research of Shanghai Jiao Tong University China Hospital Development Institute, Shanghai 200011, China; f Department of Clinical Nutrition, Zhejiang Hospital, Hangzhou 310000, China; g College of Food Science and Technology, Huazhong Agricultural University, Wuhan 430070, China; h Wuhan Puai Hospital of Tongji Medical Colledge, Huazhong University of Science and Technology, Wuhan 430034, China; i Second Provincial People's Hospital of Gansu, Lanzhou, 730000, China; j Department of Endocrinology, Changshan People's Hospital, Changshan 324200, China; k USDA Agricultural Research Service, Jean Mayer USDA Human Nutrition Research Center on Aging at Tufts University, Boston, MA, USA

**Keywords:** BMI, body mass index, HDL-C, high-density lipoprotein cholesterol, LDL-C, low-density lipoprotein cholesterol, T2D, type 2 diabetes, SNP, single-nucleotide polymorphisms, TG, triacylglycerol, TC, total cholesterol., Diabetes, Genetic variants, Interaction, Omega-3 fatty acids, Randomized controlled trial

## Abstract

**Background:**

Modulation of genetic variants on the effect of omega-3 fatty acid supplements on blood lipids is still unclear.

**Methods:**

In a double-blind randomized controlled trial, 150 patients with type 2 diabetes (T2D) were randomized into omega-3 fatty acid group (n = 56 for fish oil and 44 for flaxseed oil) and control group (n = 50) for 180 days. All patients were genotyped for genetic variants at *CD36* (rs1527483), *NOS3* (rs1799983) and *PPARG* (rs1801282). Linear regression was used to examine the interaction between omega-3 fatty acid intervention and *CD36*, *NOS3* or *PPARG* variants for blood lipids.

**Findings:**

Significant interaction with omega-3 fatty acid supplements was observed for *CD36* on triglycerides (p-interaction = 0.042) and *PPAGR* on low-density lipoprotein-cholesterol (p-interaction = 0.02). We also found a significant interaction between change in erythrocyte phospholipid omega-3 fatty acid composition and *NOS3* genotype on triglycerides (p-interaction = 0.042), total cholesterol (p-interaction = 0.013) and ratio of total cholesterol to high-density lipoprotein cholesterol (p-interaction = 0.015). The T2D patients of *CD36*-G allele, *PPARG*-G allele and *NOS3*-A allele tended to respond better to omega-3 fatty acids in improving lipid profiles. The interaction results of the omega-3 fatty acid group were mainly attributed to the fish oil supplements.

**Interpretation:**

This study suggests that T2D patients with different genotypes at *CD36*, *NOS3* and *PPARG* respond differentially to intervention of omega-3 supplements in blood lipid profiles.

## Highlights

• We replicated the interactions of *CD36*, *PPARG* and *NOS3* variants with omega-3 fatty acids in a randomized controlled trial.
• T2D patients of *CD36-G*, *PPARG-G* and *NOS3*-A allele tended to respond better to omega-3 fatty acids in improving blood lipids.

Lack of replication has become a major barrier affecting the acceleration of the field of gene-diet interaction and its translation into practice. Previous studies have suggested that genetic variants (single-nucleotide polymorphisms, SNP) at three genes (*CD36*, *NOS3* and *PPARG*) showed interaction with omega-3 fatty acids to affect the blood lipids in the intervention studies, while no replication among trials has been reported so far. The present study, with data from a well-conducted randomized controlled trial, replicated the findings of the interaction between the three genes and omega-3 fatty acids.

## 1 Introduction

There has been a pronounced progress in the field of nutrigenetics or gene-diet interaction in the past decade, thanks to the great achievement in the identification of novel genetic variants related to diseases in large-scale epidemiological studies and international consortium [[1], [2], [3], [4]]. The goal of gene-diet interaction is to tailor one's diet based on his genetic background in contrast to the traditional “one-size-fits-all” dietary recommendation. Although the concept of gene-diet interaction is appealing, and progress within recent years is encouraging, lack of replication has become a major barrier affecting the acceleration of the field and its translation into practice [1,4,5].

Omega-3 (or n-3) fatty acids, both marine (C20:5n-3, C22:5n-3, C22:6n-3) and plant based (C18:3n-3), could improve blood lipid profiles and decrease risk of cardiovascular diseases [[6], [7], [8], [9]]. Some intervention studies suggest that effects of omega-3 fatty acids on blood lipids could be modified by genetic variants and supports the existence of gene-diet interaction for omega-3 fatty acids with regard to the lipid outcomes [[10], [11], [12]]. In a systematic review, Corella et al. [13] suggested that only three genes (*CD36*, *NOS3* and *PPARG*) showed interactions with omega-3 fatty acids to affect the levels of blood lipids in the intervention studies, while no replication among trials has been reported so far.

Therefore, the aim of the present study was, to use a well-conducted randomized controlled trial to replicate the previous findings from intervention studies about the interaction of genetic variants (single-nucleotide polymorphisms, SNP) at *CD36*, *NOS3* and *PPARG* with omega-3 fatty acid intervention for the blood lipids.

## 2 Materials and Methods

### 2.1 Study Population and Design

This study was based on a double-blind randomized controlled trial. The trial was registered at ClinicalTrials.gov (No. NCT01857167), and approved by the Ethics Committee of Colledge of Biosystem Engineering and Food Science at Zhejiang University. All participants gave written informed consent.

The inclusion and exclusion criteria, and the detailed procedures of the trial has previous been reported [14]. Briefly, the inclusion criteria were fasting blood glucose>7.0 mmol/L or on use of diabetic medications, participants between 35 and 80 years for men or between postmenopausal and 80 years for women; the exclusion criteria were having familial hyperlipidemia or with blood triglycerides >4.56 mmol/L, having a history of hepatic or kidney disease or any type of cancer, or participation in another clinical trial within 30 days prior to screening. The total sample size was calculated based on a 80% power with α = 0.05 to detect a difference in HOMA-IR by 0.63 (SD 1.1) between groups, considering a 20% drop out rate [14]. A total of 185 patients with type 2 diabetes (T2D) were recruited in three research centers at Wuhan, Changshan and Lanzhou, and randomized into three groups: fish oil (n = 63), flaxseed oil (n = 61) and corn oil group (n = 61). This was a double-blind randomized controlled trial, and all the participants were randomly allocated to one of the three treatments by computer-generated random sequence. Neither the researchers randomizing the patients nor the patients knew the treatments they were allocated before the randomization or during the trial. Participants at each of the group took 4 capsules/day, corresponding to 2 g/day of C20:5n-3 and C22:6n-3 in fish oil group, and 2.5 g/day of C18:3n-3 in flaxseed oil group, with corn oil used as a control oil. The trial lasted for 180 days. All patients were asked to maintain their usual diet, lifestyle and medication, and avoid use of omega-3 fatty acid supplements. Compliance of the participants to the intervention was objectively assessed by the measurement of erythrocyte fatty acids that C20:5n-3 and C22:6n-3 was significantly increased in fish oil group compared with corn oil group, and C18:3n-3 was significantly increased in flaxseed oil group compared with corn oil group [14]. Among the trial participants, 150 patients provided DNA samples, of which 100 in omega-3 supplement group (56 in fish oil group, 44 in flaxseed oil group) and 50 in corn oil control group.

### 2.2 SNP Selection and Genotyping

SNPs rs1527483 at *CD36*, rs1799983 at *NOS3*, and rs1801282 at *PPARG* were selected for genotyping based on the prior evidence [13]. Blood DNA was isolated by using the QIAamp DNA Blood Mini Kits (Qiagen, Valenica, CA, USA). The selected SNPs were genotyped using the standard protocol recommended by the MassARRAY RS1000 (Sequenom, San Diego, CA, USA) manufacturer, and the data were analyzed by Typer 4.0 Software (Sequenom) [15], with an average genotyping success rate of 98%.

### 2.3 Measurement of Blood Lipids, Erythrocyte Phospholipid Fatty Acids, and Other Parameters

Fasting blood samples were collected at baseline and the end of the intervention. Serum high-density lipoprotein cholesterol (HDL-C), low-density lipoprotein cholesterol (LDL-C), total cholesterol (TC) and triglycerides (TG) were measured by commercially available kits with HITACHI 7020 chemistry analyser using enzyme-based colorimetric test supplied by Diasys Diagnostic Systems (Shanghai) Co., Ltd. Erythrocyte phospholipid fatty acid composition was measured by gas chromatography, as has been described previously [14]. Body weight and height were measured by trained nurses at baseline and the end of the intervention. Body mass index (BMI) was calculated as weight in kilograms divided by the square of the height in meters.

### 2.4 Statistical Analyses

All the statistical analyses were conducted using Stata (version 14; StataCorp, College Station, TX, USA). All the lipid variables (HDL-C, LDL-C, TC, TC/HDL-C, and TG) were checked for normal distribution and were natural log-transformed if not normally distributed (for TG only). Dominant models were used to assess the genetic effects and the gene-diet interactions in the present study, as to maximise the sample size in each genetic group. At baseline, the association between blood lipids and genetic variants at *CD36*, *NOS3* and *PPARG* was examined by linear regression models, adjusted for age, sex, study center and BMI.

As the primary analysis, we examined the interaction of genetic variants at *CD36*, *NOS3* and *PPARG* with omega-3 fatty acid supplements on the change in blood lipids during the intervention based on the complete case analysis. We used general linear model to test the genotype-by-intervention interaction as independent predictors of change in blood lipids, adjusting for age, sex, study center, BMI and baseline value of the corresponding outcome trait. To increase the sample size and the power to detect the interaction, we combined fish oil and flaxseed oi group into one omega-3 fatty acid supplement group, as erythrocyte phospholipid C20:5n-3 and C22:6n-3 were increased in both fish oil and flaxseed oil groups as reported previously [14]. Quanto 1.2.4 (University of Southern California) was used to estimate the detectable effect size of genotype-by-diet interactions. For example, this study had 80% power to detect significant gene-diet interaction effect sizes (for rs1527483) of 0.27 mmol/L, 0.85 mmol/L, 0.97 mmol/L, 1.05, and 0.9 mmol/L for change in HDL-C, LDL-C, TC, TC/HDL-C ratio and TG under a dominant model, respectively.

In a secondary analysis, we examined the interaction between change in erythrocyte phospholipid omega-3 fatty acids (sum of C18:3n-3, C20:5n-3, C22:5n-3 and C22:6n-3, as a continuous variable) and NOS3 genotypes for the change in lipid outcomes using the general linear model, adjusting for age, sex, study center, BMI and baseline value of the corresponding outcome trait. We conducted this secondary analysis because original paper reporting this gene-fatty acid interaction was based on the interaction between change in plasma total omega-3 fatty acids and NOS3 variant on change in TG in an intervention study [10].

If a significant interaction (p < 0.05) was detected, we conducted a stratified analysis by the genotype groups and by intervention groups using the general linear model. In addition, we also separately examined the effects of different omega-3 fatty acid group (i.e. fish oil and flaxseed oil group) on blood lipids by the tested genotypes.

In a post-hoc analysis, we generated a genetic score summing number of the omega-3 responsive allele across the 3 SNPs (rs1527483-G allele, rs1799983-A allele and rs1801282-G allele). Carriers of these alleles in the respective SNP or patients with a higher genetic score showed a better response to omega-3 fatty acids in improving lipid profiles in the present study. We examined the interaction of this genetic score (as a continuous variable) with omega-3 fatty acid intervention on blood lipids using general linear model, adjusting for age, sex, study center, BMI and baseline value of the corresponding outcome trait. We subsequently conducted stratified analysis if a significant interaction (p < 0.05) was observed.

## 3 Results

After the intervention, there were 94 patients (53 in fish oil and 41 in flaxseed oil group) left in the omega-3 fatty acid supplement group, and 45 patients in corn oil control group. The minor allele frequency of rs1527483 (A allele, *CD36*), rs1799983 (A allele, *NOS3*), and rs1801282 (G allele, *PPARG*) was 0.223, 0.073 and 0.057, respectively, and all SNPs were consistent with Hardy-Weinberg equilibrium (p > 0.05). The population characteristics by the genotypes and the intervention group were presented at Table 1. At baseline, no difference in age, BMI or lipid traits was observed among the different genotypes of the three SNPs.

**Table 1:** Baseline population characteristics by genetic variants at CD36, NOS3 and PPARG.^a^

	*CD36* (rs1527483)				*NOS3* (rs1799983)				*PPARG* (rs1801282)			
	Omega-3 supplements		Control		Omega-3 supplements		Control		Omega-3 supplements		Control	
	AA/AG (n = 41)	GG (n = 55)	AA/AG (n = 19)	GG (n = 31)	AA/AC (n = 10)	CC (n = 90)	AA/AC (n = 11)	CC (n = 39)	GG/GC (n = 12)	CC (n = 88)	GG/GC (n = 5)	CC (n = 45)
Age, y	59.2 (10.9)	60.6 (9)	59.5 (10.6)	58.1 (10.5)	62.4 (9.4)	59.8 (10)	55.5 (9.7)	59.5 (10.6)	60 (9)	60.1 (10.1)	50.8 (10.4)	59.5 (10.2)
Women, %	53.7	54.6	52.6	51.6	80	52.2	27.3	59	75	52.3	20	55.6
BMI, kg/m^2^	25.4 (3.9)	24.2 (2.9)	25.6 (3.3)	25.9 (4.8)	24.6 (1.8)	24.8 (3.6)	26.7 (7.6)	25.5 (3)	25.3 (2.9)	24.7 (3.5)	23.2 (3.4)	26.1 (4.3)
HbA1c, %	8.0 (1.9)	8.2 (2.3)	7.9 (2)	7.2 (1.2)	9.1 (2.3)	8.0 (2.1)	7.8 (2.2)	7.4 (1.4)	7.8 (1.4)	8.1 (2.2)	8.4 (2.5)	7.4 (1.4)
SBP, mmHg	142 (20)	135 (18)	141 (27)	130 (15)	136 (16)	138 (19)	124 (14)	137 (22)	135 (17)	138 (19)	120 (4)	136 (21)
DBP, mmHg	82 (9)	77 (10)	86 (20)	76 (10)	75 (9)	79 (10)	77 (7)	81 (17)	78 (11)	79 (10)	76 (8)	81 (16)
HDL-C, mmol/L	1.15 (0.31)	1.17 (0.31)	1.24 (0.21)	1.14 (0.16)	1.13 (0.3)	1.17 (0.31)	1.17 (0.15)	1.18 (0.2)	1.2 (0.28)	1.16 (0.31)	1.22 (0.13)	1.18 (0.19)
LDL-C, mmol/L	3.07 (0.78)	2.81 (0.87)	3.30 (1.02)	2.94 (0.75)	3.02 (0.69)	2.94 (0.86)	2.84 (0.54)	3.14 (0.94)	3.35 (0.77)	2.90 (0.84)	2.77 (0.58)	3.11 (0.89)
TC, mmol/L	4.84 (0.86)	4.53 (0.96)	5.17 (1.08)	4.72 (0.99)	4.72 (0.98)	4.71 (0.95)	4.66 (0.80)	4.95 (1.10)	5.23 (1.18)	4.64 (0.89)	4.60 (0.81)	4.92 (1.06)
TC/HDL-C	4.40 (1.03)	4.05 (1.12)	4.29 (1.18)	4.14 (0.79)	4.30 (0.92)	4.24 (1.13)	4.02 (0.70)	4.25 (1.00)	4.49 (0.99)	4.21 (1.13)	3.75 (0.28)	4.24 (0.98)
TG, mmol/L	1.74 (0.83)	1.71 (1.00)	1.94 (0.87)	1.81 (0.91)	1.78 (0.8)	1.73 (0.94)	1.71 (0.70)	1.90 (0.94)	2.02 (1.22)	1.70 (0.87)	1.83 (0.82)	1.86 (0.90)

BMI, body mass index; HbA1c, glycated haemoglobin; SBP, systolic blood pressure; DBP, diastolic blood pressure; HDL-C, high-density lipoprotein cholesterol; LDL-C, low-density lipoprotein cholesterol; TC, total cholesterol; TG, triglycerides.

a Values are presented as mean (SD) or as percentage.

For *CD36* SNP rs1527483, we found a significant interaction (p-interaction = 0.042) of the genotype with the intervention on serum TG levels. Omega-3 supplements marginally decreased TG levels among rs1527483-GG carriers (p = 0.067), but not among A allele carriers (p = 0.19) (Table 2). When we separated fish oil and flaxseed oil group, TG was decreased significantly among rs1527483-GG carriers after fish oil supplements (p = 0.031), but not flaxseed oil supplements (p = 0.39). No interaction was observed for other lipid outcomes.

**Table 2:** Effects of omega-3 supplements on serum lipids by different genotypes at *CD36* (rs1527483)^a^.

		Total omega-3 supplements		Fish oil supplements		Flaxseed oil supplements		Control	
		AA/AG (n = 41)	GG (n = 55)	AA/AG (n = 25)	GG (n = 29)	AA/AG (n = 16)	GG (n = 26)	AA/AG (n = 19)	GG (n = 31)
HDL-C	Baseline	1.15 (0.31)	1.17 (0.31)	1.15 (0.32)	1.14 (0.30)	1.16 (0.30)	1.21 (0.31)	1.24 (0.21)	1.14 (0.16)
	After intervention	1.20 (0.28)	1.25 (0.33)	1.24 (0.30)	1.23 (0.33)	1.14 (0.24)	1.27 (0.33)	1.37 (0.25)	1.18 (0.22)
	Change	0.08 (0.18)	0.07 (0.23)	0.10 (0.21)	0.08 (0.24)	0.04 (0.11)	0.06 (0.22)	0.15 (0.14)	0.03 (0.18)
LDL-C	Baseline	3.07 (0.78)	2.81 (0.87)	3.02 (0.78)	2.84 (0.85)	3.15 (0.81)	2.77 (0.91)	3.30 (1.02)	2.94 (0.75)
	After intervention	2.86 (0.71)	2.72 (0.81)	2.77 (0.69)	2.57 (0.89)	3.00 (0.74)	2.89 (0.69)	3.26 (0.97)	2.92 (0.90)
	Change	−0.23 (0.77)	−0.10 (0.77)	−0.3 (0.89)	−0.3 (0.81)	−0.12 (0.54)	0.12 (0.68)	−0.13 (0.99)	0.01 (0.70)
TC	Baseline	4.84 (0.86)	4.53 (0.96)	4.82 (0.88)	4.47 (0.93)	4.85 (0.86)	4.61 (1.00)	5.17 (1.08)	4.72 (0.99)
	After intervention	4.77 (0.86)	4.63 (0.99)	4.66 (0.80)	4.41 (1.05)	4.96 (0.95)	4.87 (0.88)	5.23 (1.08)	5.00 (1.22)
	Change	−0.05 (0.81)	0.06 (0.81)	−0.19 (0.84)	−0.12 (0.72)	0.18 (0.74)	0.26 (0.87)	0.22 (1.14)	0.29 (0.89)
TC/HDL-C	Baseline	4.4 (1.03)	4.05 (1.12)	4.40 (0.98)	4.09 (1.13)	4.40 (1.13)	3.99 (1.14)	4.29 (1.18)	4.14 (0.79)
	After intervention	4.13 (1.02)	3.91 (1.14)	3.90 (0.86)	3.80 (1.16)	4.50 (1.17)	4.03 (1.14)	3.91 (0.85)	4.43 (1.87)
	Change	−0.34 (0.64)	−0.16 (0.87)	−0.56 (0.58)	−0.34 (0.72)	0.02 (0.60)	0.03 (0.98)	−0.42 (0.87)	0.31 (1.84)
TG (log-transformed)	Baseline	0.44 (0.5)	0.40 (0.51)	0.45 (0.50)	0.39 (0.43)	0.42 (0.52)	0.43 (0.60)	0.56 (0.47)	0.48 (0.49)
	After intervention	0.39 (0.52)	0.28 (0.54)	0.27 (0.49)	0.20 (0.51)	0.59 (0.53)	0.36 (0.57)	0.37 (0.56)	0.54 (0.42)
	Change⁎	−0.07 (0.34)	−0.13 (0.42)	−0.18 (0.35)	−0.18 (0.39)	0.10 (0.25)	−0.07 (0.45)	−0.21 (0.37)	0.01 (0.33)

HDL-C, high-density lipoprotein cholesterol; LDL-C, low-density lipoprotein cholesterol; TC, total cholesterol; TG, triglycerides.

a Values are presented as mean (SD). Total omega-3 supplement group is a combination of fish oil and flaxseed oil groups.

⁎ Significant interaction (p-interaction = 0.042) between the *CD36* genotypes and the total omega-3 supplements for the change in TG was observed in the general linear model, after adjustment for age, sex, study center, BMI and baseline value corresponding outcome. *CD36* GG allele carriers showed a decrease in TG after intervention of omega-3 supplements (p = 0.067), especially in the fish oil group (p = 0.031) (compared with control group), but not in the A allele carriers.

We did not find any significant interaction between *NOS3* SNP rs1799983 and omega-3 fatty acid supplements on lipid traits (Table 3). In our secondary analysis, we found that change in erythrocyte phospholipid omega-3 fatty acids had significant interaction with rs1799983 on serum TG (p-interaction = 0.042), TC (p-interaction = 0.013) and TC/HDL-C (p-interaction = 0.015) (Fig. 2). In the low omega-3 fatty acid change group (<1.38%, calculated based on the median level of the omega-3 fatty acid change), rs1799983 A allele carriers had increased change in TG (p = 0.035), TC (p = 0.02) and TC/HDL-C (p = 0.035) compared with CC carriers, while no difference was found in the high omega-3 fatty acid change group (≥1.38%).

**Fig. 1:**
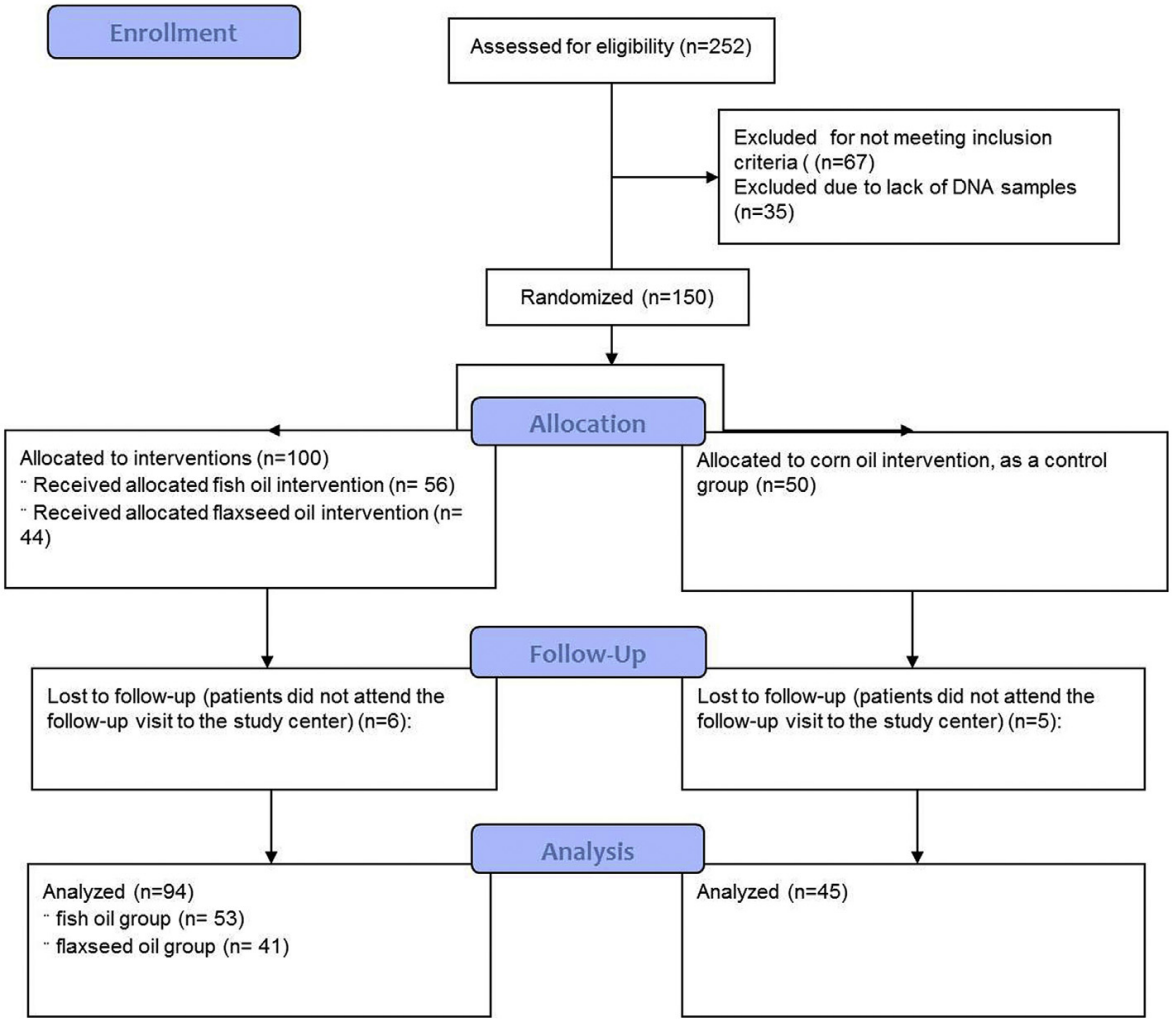
Flow diagram of the participants in the randomized controlled trial.

**Table 3:** Effects of omega-3 supplements on serum lipids by different genotypes at *NOS3* (rs1799983).^a^

		Total omega-3 supplements		Fish oil supplements		Flaxseed oil supplements		Control	
		AA/AC (n = 10)	CC (n = 90)	AA/AG (n = 3)	GG (*n* = 53)	AA/AG (*n* = 7)	GG (*n* = 37)	AA/AC (n = 11)	CC (n = 39)
HDL-C	Baseline	1.13 (0.30)	1.17 (0.31)	1.38 (0.37)	1.13 (0.30)	1.03 (0.21)	1.21 (0.32)	1.17 (0.15)	1.18 (0.20)
	After intervention	1.32 (0.29)	1.21 (0.31)	1.64 (0.33)	1.21 (0.29)	1.19 (0.13)	1.22 (0.33)	1.22 (0.19)	1.26 (0.26)
	Change	0.19 (0.12)	0.06 (0.22)	0.26 (0.14)	0.08 (0.23)	0.16 (0.11)	0.03 (0.19)	0.09 (0.10)	0.07 (0.19)
LDL-C	Baseline	3.02 (0.69)	2.94 (0.86)	3.24 (1.02)	2.95 (0.83)	2.93 (0.58)	2.94 (0.92)	2.84 (0.54)	3.14 (0.94)
	After intervention	2.87 (0.75)	2.79 (0.78)	2.14 (0.31)	2.71 (0.82)	3.19 (0.66)	2.91 (0.71)	2.92 (0.63)	3.08 (1.00)
	Change	−0.15 (1.08)	−0.17 (0.77)	−1.10 (0.96)	−0.27 (0.86)	0.26 (0.9)	−0.02 (0.59)	0.19 (0.93)	−0.11 (0.78)
TC	Baseline	4.72 (0.98)	4.71 (0.95)	5.38 (1.45)	4.66 (0.94)	4.44 (0.65)	4.77 (0.97)	4.66 (0.80)	4.95 (1.10)
	After intervention	4.96 (1.13)	4.71 (0.95)	4.13 (0.41)	4.60 (1.01)	5.32 (1.17)	4.86 (0.83)	4.54 (0.75)	5.23 (1.22)
	Change	0.24 (1.39)	−0.02 (0.75)	−1.25 (1.04)	−0.11 (0.76)	0.88 (0.97)	0.13 (0.72)	0.06 (1.12)	0.32 (0.94)
TC/HDL-C	Baseline	4.30 (0.92)	4.24 (1.13)	3.93 (0.71)	4.30 (1.11)	4.46 (1.00)	4.15 (1.18)	4.02 (0.70)	4.25 (1.00)
	After intervention	3.90 (1.12)	4.07 (1.14)	2.58 (0.48)	3.95 (1.01)	4.46 (0.75)	4.26 (1.30)	3.75 (0.65)	4.37 (1.73)
	Change	−0.40 (1.03)	−0.21 (0.86)	−1.36 (0.32)	−0.42 (0.78)	0.00 (0.96)	0.09 (0.89)	−0.24 (0.83)	0.13 (1.74)
TG (log-transformed)	Baseline	0.48 (0.46)	0.42 (0.51)	0.65 (0.41)	0.42 (0.47)	0.41 (0.49)	0.43 (0.57)	0.45 (0.46)	0.53 (0.49)
	After intervention	0.40 (0.43)	0.33 (0.54)	0.19 (0.46)	0.26 (0.51)	0.49 (0.41)	0.43 (0.58)	0.47 (0.44)	0.47 (0.50)
	Change	−0.08 (0.31)	−0.11 (0.39)	−0.46 (0.15)	−0.16 (0.37)	0.08 (0.19)	−0.03 (0.42)	−0.02 (0.31)	−0.08 (0.37)

HDL-C, high-density lipoprotein cholesterol; LDL-C, low-density lipoprotein cholesterol; TC, total cholesterol; TG, triglycerides. No interaction between *NOS3* genotypes and omega-3 supplements for the lipid traits was observed.

a Values are presented as mean (SD). Total omega-3 supplement group is a combination of fish oil and flaxseed oil groups.

For *PPARG* SNP rs1801282, we observed that omega-3 supplements interacted with the SNP to modulate LDL-C levels (p-interaction = 0.02). Stratified analysis suggested that GG/GC allele carriers had a significantly higher increase in LDL-C compared with CC carriers in the control group (p = 0.022), but no difference was observed in the total omega-3 group, fish oil or flaxseed oil group (Table 4).

**Table 4:** Effects of omega-3 supplements on serum lipids by different genotypes at *PPARG* (rs1801282)^a^.

		Total omega-3 supplements		Fish oil supplements		Flaxseed oil supplements		Control	
		GG/GC (n = 12)	CC (n = 88)	AA/AG (n = 8)	GG (*n* = 48)	AA/AG (n = 4)	GG (*n* = 40)	GG/GC (n = 5)	CC (n = 45)
HDL-C	Baseline	1.20 (0.28)	1.16 (0.31)	1.16 (0.31)	1.14 (0.31)	1.26 (0.26)	1.17 (0.31)	1.22 (0.13)	1.18 (0.19)
	After intervention	1.28 (0.25)	1.22 (0.31)	1.26 (0.25)	1.23 (0.32)	1.34 (0.31)	1.20 (0.31)	1.31 (0.12)	1.25 (0.26)
	Change	0.08 (0.27)	0.07 (0.21)	0.10 (0.29)	0.09 (0.22)	0.03 (0.22)	0.05 (0.19)	0.07 (0.13)	0.08 (0.18)
LDL-C	Baseline	3.35 (0.77)	2.90 (0.84)	3.19 (0.86)	−0.26 (0.85)	3.67 (0.51)	0.05 (0.66)	2.77 (0.58)	3.11 (0.89)
	After intervention	2.76 (0.68)	2.80 (0.79)	2.53 (0.63)	2.92 (0.83)	3.36 (0.39)	2.86 (0.87)	3.47 (0.54)	3.00 (0.96)
	Change⁎	−0.54 (0.87)	−0.12 (0.78)	−0.65 (0.98)	2.71 (0.84)	−0.23 (0.47)	2.92 (0.71)	0.80 (0.68)	−0.13 (0.78)
TC	Baseline	5.23 (1.18)	4.64 (0.89)	4.92 (1.18)	4.66 (0.94)	5.85 (1.05)	4.60 (0.85)	4.60 (0.81)	4.92 (1.06)
	After intervention	4.91 (1.03)	4.71 (0.96)	4.66 (1.15)	4.55 (0.98)	5.49 (0.26)	4.90 (0.91)	5.27 (0.79)	5.07 (1.20)
	Change	−0.43 (0.94)	0.07 (0.81)	−0.43 (0.99)	−0.14 (0.79)	−0.42 (1.02)	0.31 (0.78)	0.61 (0.80)	0.23 (0.99)
TC/HDL-C	Baseline	4.49 (0.99)	4.21 (1.13)	4.38 (1.12)	4.26 (1.10)	4.71 (0.77)	4.15 (1.17)	3.75 (0.28)	4.24 (0.98)
	After intervention	3.97 (1.06)	4.06 (1.14)	3.86 (1.15)	3.87 (1.03)	4.25 (0.96)	4.30 (1.24)	4.04 (0.49)	4.27 (1.66)
	Change	−0.55 (1.24)	−0.19 (0.83)	−0.63 (1.51)	−0.45 (0.65)	−0.35 (0.07)	0.11 (0.92)	0.29 (0.41)	0.03 (1.67)
TG (log-transformed)	Baseline	0.56 (0.54)	0.41 (0.49)	0.42 (0.49)	0.43 (0.46)	0.85 (0.59)	0.39 (0.54)	0.49 (0.57)	0.51 (0.47)
	After intervention	0.42 (0.43)	0.32 (0.54)	0.32 (0.47)	0.24 (0.51)	0.67 (0.12)	0.42 (0.56)	0.81 (0.36)	0.44 (0.48)
	Change	−0.17 (0.41)	−0.10 (0.38)	−0.10 (0.37)	−0.19 (0.37)	−0.35 (0.55)	0.02 (0.37)	0.10 (0.20)	−0.09 (0.37)

HDL-C, high-density lipoprotein cholesterol; LDL-C, low-density lipoprotein cholesterol; TC, total cholesterol; TG, triglycerides.

a Values are presented as mean (SD). Total omega-3 supplement group is a combination of fish oil and flaxseed oil groups.

⁎ Significant interaction (p-interaction = 0.02) between the *PPARG* genotypes and the omega-3 supplements for the change in LDL-C was observed in the general linear model, after adjustment for age, sex, study center, BMI and baseline value of corresponding outcome. *PPARG* G allele carriers had a higher increase in LDL-C level compared with CC carriers among control group (p = 0.022), while no difference was observed among omega-3 groups.

We observed a significant interaction between the genetic score and omega-3 fatty acid supplements on TG levels (p-interaction = 0.04) (Fig. 3), not for other lipids. Among the control group, serum TG levels were significantly higher (p = 0.008) in high genetic score group (compared with low genetic score group), while no difference was observed among omega-3 supplement group. Omega-3 supplements significantly decreased serum TG levels compared with control only among participants with a high genetic score (p = 0.026), and only fish oil (p = 0.009), not flaxseed oil, decreased TG in the subgroup analysis. (See Fig. 3).

**Fig. 2:**
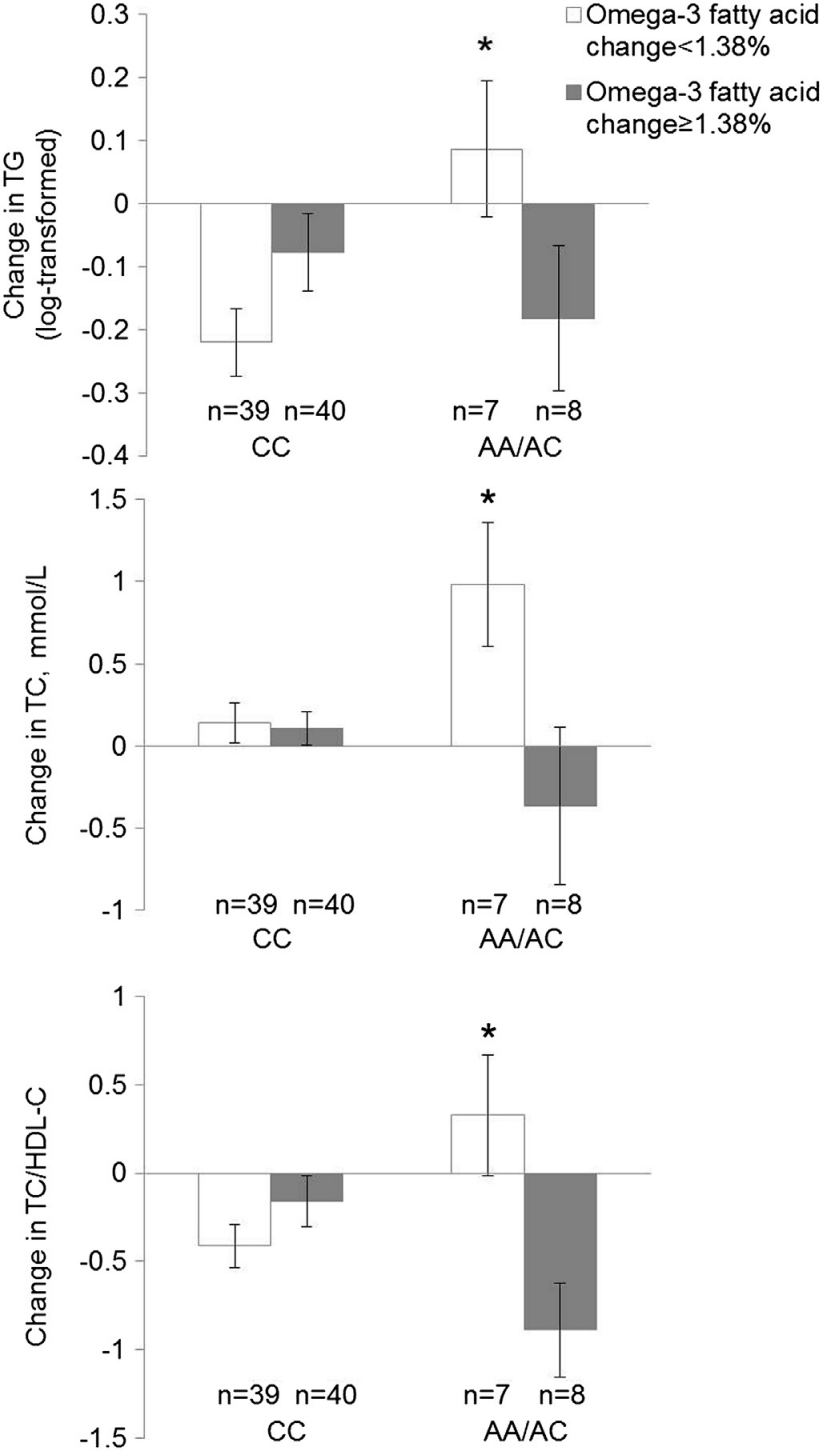
Change in serum lipids stratified by change in erythrocyte phospholipid omega-3 fatty acids and *NOS3* rs1799983 during the intervention. Values and error bars are presented as mean and standard error. General linear model was used to estimate the interaction between the change in erythrocyte phospholipid omega-3 fatty acids and rs1799983 for the change in serum lipids, with significant interaction observed for TG (p = 0.042), TC (p = 0.013) and TC/HDL-C (p = 0.015). *p < 0.05 indicated significant difference in the lipid change between different genotype carriers among the low omega-3 fatty acid change group (<1.38%, calculated based on the median level of the omega-3 fatty acid change). HDL-C, high-density lipoprotein cholesterol; TC, total cholesterol; TG, triglycerides.

**Fig. 3:**
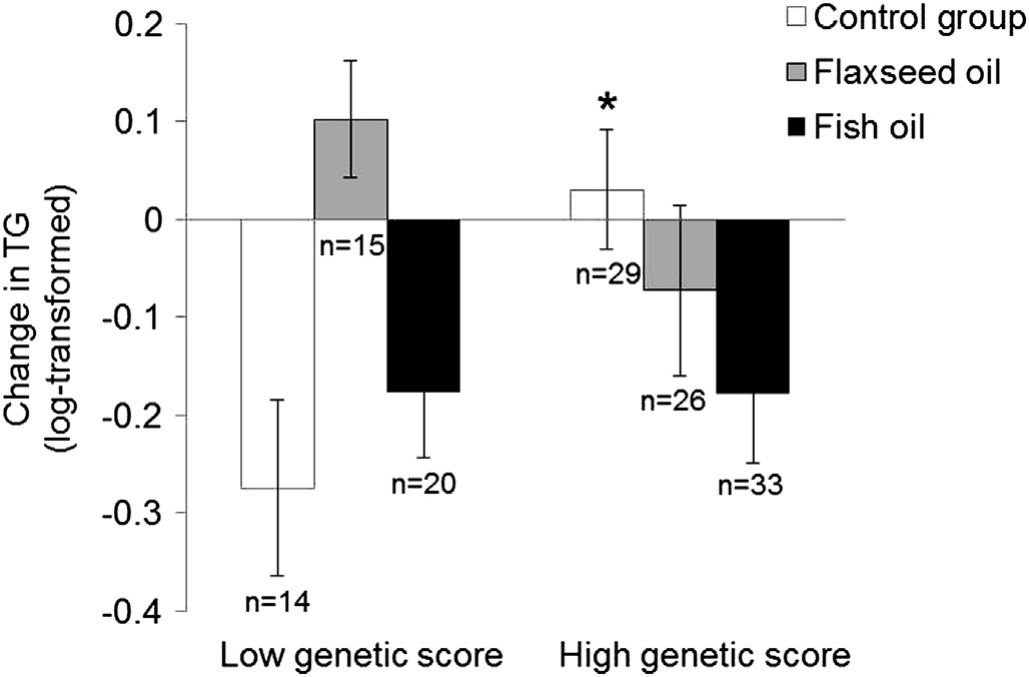
Change in serum triglycerides stratified by omega-3 fatty acid supplements and genetic score of omega-3 responsive alleles. Values and error bars are presented as mean and standard error. General linear model was used to estimate the interaction between the total omega-3 fatty acid supplements and a genetic score of omega-3 responsive variants for the change in serum triglycerides (TG, p = 0.04). The genetic score was a sum of the omega-3 responsive allele across the 3 SNPs (rs1527483-G allele, rs1799983-A allele and rs1801282-G allele). Carriers of these alleles in the respective variant showed a better response to omega-3 fatty acids in terms of improved lipid profiles in the present study. High genetic score: score 2–4; low genetic score: score 0–1. *p < 0.05 indicated significant difference in the TG change between different genetic score group among the control group and no significant difference was observed in the other two groups.

## 4 Discussion

In the present study, we successfully replicated the interaction of genetic variants at *CD36*, *NOS3* and *PPARG* with omega-3 fatty acids on blood lipids. The T2D patients with *CD36* major allele GG genotype, but not A allele carriers, displayed a decreased TG concentration in response to the omega-3 intervention. Carriers of the *PPARG* minor G allele, compared with those carrying CC genotype, showed increased levels of LDL-C among control group, but not among the omega-3 intervention group. We also replicated the interaction between erythrocyte omega-3 fatty acid change and *NOS3* variant on blood lipids that rs1799983 minor A allele carriers responded better to high erythrocyte omega-3 fatty acid in improving lipid profiles. A genetic score generated based on the three SNPs demonstrated a combined effects of the three SNPs for their interactions with omega-3 supplements on blood lipids. The effect of the omega-3 supplement group was mainly attributed to the fish oil supplements.

In a prior intervention study, 111 healthy Caucasian men took 1.71 g omega-3 fatty acids (C20:5n-3 + C22:6n-3) per day for 12 weeks [12], significant decrease in TG only occurred in participants with the GG variant of several *CD36* SNPs, including rs1527483 [12]. CD36 is a gene encoding CD36 protein (also known as fatty acid translocase [FAT]). CD36 binds long-chain fatty acids and facilitates its transfer into cell, playing an important role in fatty acid metabolism [[16], [17], [18], [19]]. A haplotype containing the rs1527483 G allele was associated with increased levels of TG, and was potentially associated with CD36 deficiency, which led to the decrease in free fatty acid clearance and increase in the hepatic free fatty acid update and production of TG [20]. It is also known that omega-3 fatty acids, as PPAR agonist, could induce the *CD36* mRNA expression in different cell types [21,22], which may be potentially through the regulation of the PPARG activity [[23], [24], [25]].

Therefore, the effects of omega-3 fatty acids on TG may partly involve the modulation of CD36 activity, which renders the interaction between *CD36* variants and omega-3 intervention biologically plausible. Based on the prior evidence [12] and results from the present study, it could be hypothesized that TG-lowering effects of omega-3 supplements may be more evident in the rs1527483 GG carriers, as a lower CD36 activity in the GG group is more likely to be increased/restored by the omega-3 supplements compared to the other genotype group. The functional SNPs in *CD36* gene responsible for the observed interaction are still not clear and warrant further investigation.

*NOS3* gene encodes nitric oxide synthase 3, metabolizing l-arginine to nitric oxide. Homozygotes (AA carriers) of the *NOS3* SNP rs1799983 (also known as Glu298Asp) A minor allele have increased risk [26] of cardiovascular diseases [10]. In a randomized trial among 450 individuals with metabolic syndrome, carriers of the rs1799983 A allele showed a greater response to increased plasma omega-3 fatty acid levels in terms of reduction in TG levels [10]. In a cross-sectional analysis of 248 participants, increased omega-3 fatty acid levels were positively associated with endothelial function only in rs1799983 A allele carriers, but not in the CC genotype carriers [27]. In another postprandial study among 30 rs1799983 A allele carriers and 29 rs1799983 C allele carriers, only women with rs1799983 A allele were responsive to the beneficial effect of omega-3 fatty acids on endothelial function [28].

The above literature together with the findings from the present study suggest that rs1799983 minor A allele carriers might achieve more benefits in terms of improved lipids and other cardiovascular profiles in response to a higher omega-3 fatty acid exposure, compared to those with CC genotype. The above observed interaction is biological plausible. The minor A allele was suggested to be associated with a lower NOS3 activity and lower NOS3 protein enrichment in the caveolar membrane fraction [29], while inhibition of NOS was found to be associated with an increase in circulating TG and cholesterol [29]. Dietary omega-3 fatty acid supplements could increase basal endothelial NO production and also increase NOS3 mRNA and protein levels [29]. Moreover, omega-3 fatty acids were shown to regulate caveolar microenvironment, including the distribution and translocation of NOS in caveolar [30,31]. Therefore, it is possible that omega-3 fatty acid supplements compensate the disrupted NOS3 activity caused by the rs1799983 minor A allele, whereas the homozygotes of the C major allele might not get additional benefit given its normal NOS3 activity in caveolar.

*PPARG* encodes peroxisome proliferator-activated receptor-gamma, which is a transcription factor that regulates various genes involved in lipid storage, adipogenesis and insulin sensitization [32]. Animal model showed that the rs1801282 variant, as an important modulator in metabolic control, was strongly subject to the influence of dietary factors or gene-diet interaction [33]. This concept was further confirmed in human studies, where the interaction of the rs1801282 with dietary fat intake has been reported in various populations [11,[34], [35], [36], [37], [38]]. In a controlled trial among 150 individuals, participants were randomized to consume fish oil supplement or placebo oil for 3 months. In that trial, rs1801282 G allele carriers had a greater decrease in serum TG in response to the omega-3 fatty acid supplements, compared with the CC carriers [11]. SNP rs1801282 G allele carriers had higher levels of LDL-C compared with CC carriers in a previous meta-analysis among Asian populations [39]. We found consistent association in the control group, but not in the omega-3 group. We hypothesized that the deleterious effect of rs1801282 G allele on LDL-C was attenuated by omega-3 fatty acids supplements.

There are several limitations in the present study. First, the sample size of the present study is moderate, limiting the statistical power of detecting a gene-diet interaction. Second, the combined intervention group has a double sample size than the control group. However, the impact of the difference in sample size on the interaction analysis should be minimal, as we have also examined the interaction for fish oil and flaxseed oil separately compared with control group and the results of fish oil is consistent with the combined intervention group across different tested genes. Third, potential false positive results may occur due to multiple testing, although we intends to replicate the gene-diet interaction in previous reports and the tests are hypothesis driven. We further demonstrate the existence of the interaction by using a generic score of the 3 tested SNPs. Fourth, our study is based on a Chinese population with T2D and the generalizability to other ethnicities or healthy populations may be limited. The major strength of the present study is that it is based on a well-conducted double-blind randomized controlled trial, which lasts for 180 days with good participant compliance as measured by erythrocyte fatty acid composition.

In conclusions, using a double-blind randomized controlled trial, we replicated the interaction between omega-3 fatty acids and genetic variants at *CD36, NOS3* and *PPARG* on blood lipids reported in previous intervention study. These replications suggest that the effects of omega-3 fatty acids on blood lipids may vary by genetic variation at *CD36, NOS3, PPARG* genes, and a personalized diet recommendation based on certain genetic make-up to improve blood lipid profiles may work specifically for omega-3 fatty acid intake. Nonetheless, this study is still quite preliminary, and more trials with larger sample size are warrant.
